# Association of Continuous Glucose Monitoring Use and Hemoglobin A_1c_ Levels Across the Lifespan Among Individuals With Type 1 Diabetes in the US

**DOI:** 10.1001/jamanetworkopen.2022.23942

**Published:** 2022-07-27

**Authors:** Joshua M. Weinstein, Anna R. Kahkoska

**Affiliations:** 1 Department of Health Policy and Management, Gillings School of Global Public Health, University of North Carolina, Chapel Hill; 2 Department of Nutrition, University of North Carolina, Chapel Hill

## Abstract

This cross-sectional study investigates age, probability of continuous glucose monitoring use, and their association with glycemic control across the lifespan among US individuals with type 1 diabetes.

## Introduction

Type 1 diabetes (T1D) affects individuals of all ages, including a growing population of older adults.^1^ Small, remote continuous glucose monitoring (CGM) devices^2,3^ are now recommended as the standard for glucose monitoring for T1D.^2^ However, few studies have examined age, the probability of CGM use, and the association of CGM use with glycemic control across the lifespan.

## Methods

This cross-sectional study used data from the national T1D Exchange Registry (2017-2018) for individuals aged 10 to 85 years. This registry includes data from patients with T1D at 80 US clinics nationwide,^4^ which are collected through medical record extraction and patient questionnaires. The University of North Carolina Institutional Review Board deemed this study exempt from review owing to the use of deidentified data. Informed consent was obtained from each participant (or by a parent or guardian if aged <18 years) by the original investigators. The study followed the STROBE reporting guideline.

We fit a generalized additive model^5^ estimating the probability of CGM use with age specified as a penalized spline, and we controlled for health insurance, sex, annual household income, race and ethnicity, education level, and insulin delivery method. We included race and ethnicity variables owing to well-documented disparities in diabetes treatments and outcomes among Black and Hispanic individuals. We present results as the fitted probability of CGM use across age. We then fit a linear regression model estimating hemoglobin A_1c_ (HbA_1c_) levels by CGM use, age, age squared, and interaction of CGM use and age, controlling for the same covariates as described. We used the inverse probability of treatment weights and included the same covariates in both the propensity score and the HbA_1c_ outcome models for a doubly robust approach. We imputed missing covariates for HbA_1c_ modeling using multivariate imputation via chained equations.

We used 2-sided hypothesis tests with a significance threshold of *P* < .05. Data were modeled using R version 4.1.0 (R Foundation for Statistical Computing) and Stata 16.1 (StataCorp).

## Results

Our sample included 19 261 patients aged 10 to 85 years (mean [SD] of 27.58 [17.65 ] years) with T1D (Table). Of these individuals, 9745 (50.59%) were female and 9478 (49.21%) were male; sex was unknown for 38 (0.20%). The mean (SD) HbA_1c_ level was 8.57% (1.81%) (to convert to a proportion of total hemoglobin, multiply by 0.01), and 5779 patients (30.00%) reported CGM use.

**Table.** Descriptive Statistics of the Analytic Sample From the Type 1 Diabetes Exchange Registry

Characteristic	Patients^a^		
	Overall (N = 19 261)	By CGM use	
		Nonusers (n = 13 482)	Users (n = 5779)
Age, mean (SD), y	27.58 (17.65)	26.64 (17.36)	29.77 (18.13)
Hemoglobin A_1c_, mean (SD)^b^	8.57 (1.81)	8.86 (1.89)	7.90 (1.37)
Sex			
Female	9745 (50.59)	6723 (49.87)	3022 (52.29)
Male	9478 (49.21)	6733 (49.94)	2745 (47.50)
Unknown	38 (0.20)	26 (0.19)	12 (0.21)
Race and ethnicity^c^			
American Indian or Alaska Native	84 (0.44)	70 (0.52)	14 (0.24)
Asian	205 (1.06)	140 (1.04)	65 (1.12)
Black non-Hispanic	1045 (5.43)	945 (7.01)	100 (1.73)
Hispanic or Latino	1563 (8.11)	1258 (9.33)	305 (5.28)
Native Hawaiian or other Pacific Islander	27 (0.14)	24 (0.18)	3 (0.05)
White non-Hispanic	15 696 (81.49)	10 555 (78.29)	5141 (88.96)
Multiple races or ethnicities	518 (2.69)	401 (2.97)	117 (2.02)
Unknown	123 (0.64)	89 (0.66)	34 (0.59)
Health insurance			
Private	13 813 (71.71)	8936 (66.28)	4877 (84.39)
Public or single service	4458 (23.15)	3778 (28.02)	680 (11.77)
Uninsured	196 (1.02)	167 (1.24)	29 (0.50)
Unknown	794 (4.12)	601 (4.46)	193 (3.34)
Annual household income, $			
≤35 000	2608 (13.54)	2210 (16.39)	398 (6.89)
35 001-50 000	1634 (8.48)	1267 (9.40)	367 (6.35)
50 001-75 000	2378 (12.35)	1677 (12.44)	701 (12.13)
≥75 001	7431 (38.58)	4470 (33.16)	2961 (51.24)
Unknown	5210 (27.05)	3858 (28.62)	1352 (23.40)
Educational attainment			
Less than high school	930 (4.83)	776 (5.76)	154 (2.66)
High school diploma	2295 (11.92)	1900 (14.09)	395 (6.84)
Some college but no degree	3458 (17.95)	2709 (20.09)	749 (12.96)
Associate degree	1961 (10.18)	1442 (10.70)	519 (8.98)
Bachelor’s degree	5159 (26.78)	3230 (23.96)	1929 (33.38)
Graduate, doctorate, or professional degree	3977 (20.65)	2280 (16.91)	1697 (29.36)
Unknown	1481 (7.69)	1145 (8.49)	336 (5.81)
Insulin modality			
Combination insulin pump and manual daily injections	220 (1.14)	145 (1.08)	75 (1.30)
Insulin pump	11 976 (62.18)	7217 (53.53)	4759 (82.35)
Manual daily injections	7026 (36.48)	6089 (45.16)	937 (16.21)
Unknown	39 (0.20)	31 (0.23)	8 (0.14)

Abbreviation: CGM, continuous glucose monitoring.

^a^ Unless noted otherwise, data are presented as No. (%) of patients. Percentages have been rounded and therefore may not total 100.

^b^ To convert to a proportion of total hemoglobin, multiply by 0.01.

^c^ The T1D Exchange Registry collects participant data through medical record extraction and questionnaires administered at care visits. Clinic staff contacted patients by phone to ascertain missing information. It is likely that some of the race and ethnicity data were obtained through self-report, whereas others may have been obtained through clinician notes in medical records.

The adjusted probability of CGM use decreased in adolescence, increased afterward until approximately age 40, remained relatively constant until age 60, and then decreased until age 75 (Figure). CGM use was associated with lower HbA_1c_ levels across age compared with nonuse, but this association waned with increased age. The adjusted mean difference in HbA_1c_ levels among CGM users relative to nonusers was −0.70% at age 10 years (ie, 0.70% lower among users), which decreased to −0.62% at 20, −0.55% at 30, −0.48% at 40, −0.41% at 50, −0.34% at 60, −0.27% at 70, −0.20% at 80, and −0.16 at 85 years.

**Figure.**
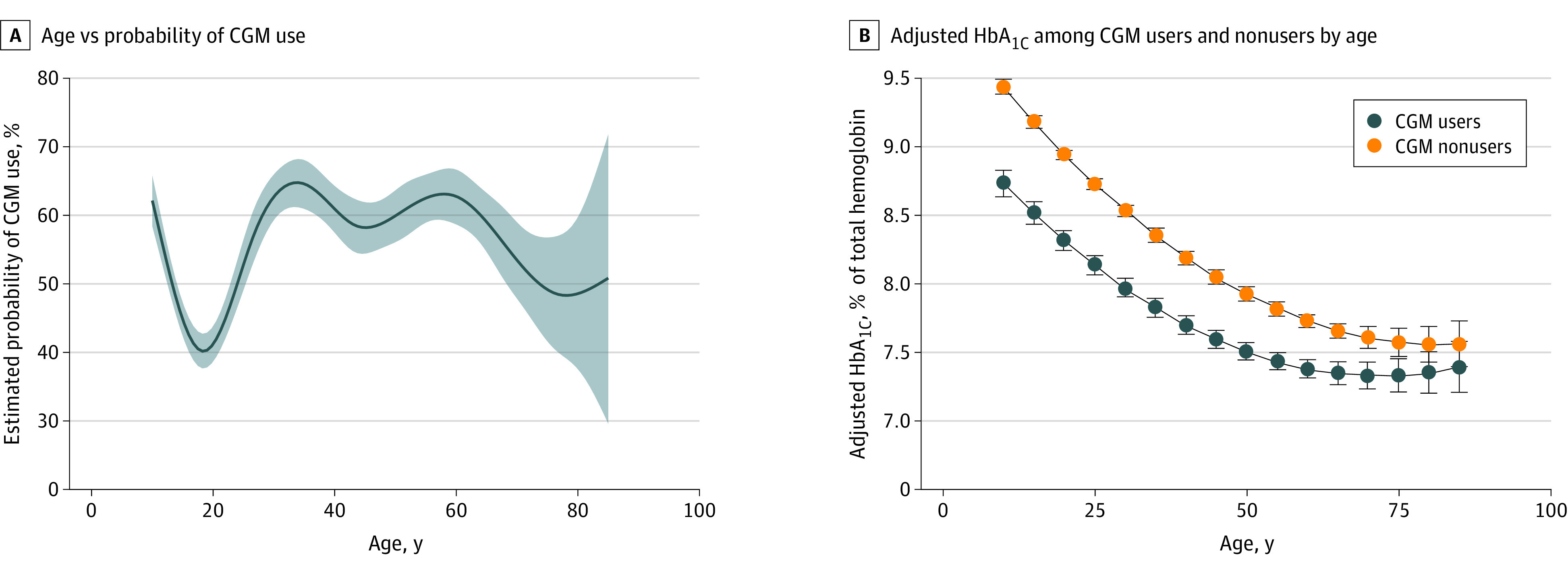
Age and Probability of Continuous Glucose Monitoring (CGM) Use and Effects on Hemoglobin A_1c_ (HbA_1c_) Levels To convert HbA_1c_ levels to a proportion of total hemoglobin, multiply by 0.01.

## Discussion

This cross-sectional study found that CGM use varied across age, with the highest adjusted probability occurring in middle adulthood. Furthermore, the probability of CGM utilization decreased with increasing age in older adulthood, reflecting the barriers that Medicare patients face regarding CGM coverage. The clinically significant differences in HbA_1c_ levels (>0.50%) among CGM users and nonusers among youth and adult populations underscore the need to identify age-related barriers to CGM use. Decreases in HbA_1c_ differences over the lifespan suggest that adolescence is partly associated with higher HbA_1c_ levels^6^ as well as possible survivorship bias, because older individuals with T1D may have better glycemic control regardless of CGM use. There are likely benefits to CGM use among older adults not reflected in the HbA_1c_ outcome (eg, reduced hypoglycemia).^1^

Limitations of this study include the cross-sectional design, which precludes causal inference. In addition, the predominance of non-Hispanic White individuals limits the generalizability of the findings to other racial and ethnic groups. Future work should explore patterns in CGM use and severe hypoglycemia, diabetic ketoacidosis, and health care utilization over the lifespan.
